# Autophagy Defects and Mitochondrial Retention in Erythroid Diseases

**DOI:** 10.3390/ijms27198467

**Published:** 2026-09-23

**Authors:** Claudio M. Fader, Julieta Jacob, Santiago Castro Perez, Cécile Deleschaux, Alice Dussouchaud, Gaël Nicolas, Betiana N. Salassa, Sophie D. Lefevre, Mariano A. Ostuni

**Affiliations:** 1 Laboratorio de Biología Celular y Molecular, Instituto de Histología y Embriología (IHEM), Universidad Nacional de Cuyo, CONICET, Mendoza M5502, Argentina; julietajacobg@gmail.com (J.J.); castroperez.santiago@uncuyo.edu.ar (S.C.P.); nsalassa@fodonto.uncu.edu.ar (B.N.S.); 2 Facultad de Odontología, Universidad Nacional de Cuyo, Mendoza M5502, Argentina; 3 Facultad de Medicina, Universidad del Aconcagua, Mendoza M5502, Argentina; 4 Université Paris Cité, INSERM, EFS, BIGR U1134, team PAMS, 75015 Paris, France; cecile.deleschaux@inserm.fr (C.D.); alice.dussouchaud@inserm.fr (A.D.); gael.nicolas@inserm.fr (G.N.); sophie.lefevre@inserm.fr (S.D.L.); 5 Initiatives IdEx Globule Rouge d’Excellence (InIdex GR-Ex), Université Paris Cité, 75015 Paris, France

**Keywords:** autophagy, erythropoiesis, hemoglobinopathies, mitophagy, anemia

## Abstract

Erythropoiesis requires extensive cellular remodeling, culminating in enucleation and organelle clearance to generate mature red blood cells (RBCs). Over the past 15 years, accumulating evidence has established that macroautophagy—particularly ULK1-, NIX-, and PINK1-dependent mitophagy—plays a central role in erythroid maturation. Defective autophagy can lead to mitochondrial retention, increased reactive oxygen species (ROS) production, membrane alterations, and ineffective erythropoiesis. Such abnormalities have now been documented in several hematological disorders, including myelodysplastic syndromes, Fanconi anemia, Diamond–Blackfan anemia, sickle cell disease, and β-thalassemia. This review summarizes current knowledge of autophagy regulation during erythropoiesis, the molecular mechanisms governing mitochondrial clearance, and the pathological consequences of impaired autophagy in red blood cell disorders. Finally, we discuss emerging therapeutic strategies targeting autophagy pathways.

## 1. Introduction: Erythropoiesis and Cellular Remodeling

With a production rate of 2.3 million cells per second, red blood cells (RBCs) account for the most proliferative tissue of the human body (reviewed in [1]). Erythropoiesis has then to be tightly controlled as even minor changes in kinetics lead to major changes in RBC production. Flawless erythropoiesis is also required to produce fully functional RBCs, whose lifespans contribute to maintaining hematocrit. Erythropoiesis is defined by three essential phases: (1) the progenitors’ commitment, a highly proliferative phase driving the yield of RBC production; (2) the precursors’ specialization, characterized by a reduced proliferative capacity and heavy morphological changes; and (3) the reticulocyte maturation, leading to the acquisition of peculiar features such as the RBC biconcave form and its high deformability. Throughout terminal erythropoiesis, precursors, called erythroblasts, and reticulocytes undergo significant changes, such as a complete proteome modification, which includes massive hemoglobin production, remodeling of the cell membrane and cytoskeleton, and the elimination of organelles [2,3] (Figure 1).

The transition between progenitors and precursors is regulated by specific cytokines, mainly erythropoietin, and iron availability. From this step, proteome changes are initiated by the activation of a specific transcriptional process, under the control of the major erythropoietic transcription factor GATA1. As for proteome remodeling, organelle clearance is a continuous process that occurs throughout all stages of erythroblasts maturation. During this process, organelles play a dual role: they must remain fully functional to support the intense biosynthetic and metabolic demands of differentiating erythroblasts, while being progressively eliminated as maturation proceeds (reviewed in [4]). A striking example would be mitochondrial clearance. Functional mitochondria are essential to produce the large amount of heme that is required for hemoglobin synthesis, but at the same time, mitochondria need to be cleared as mature RBCs are devoid of this organelle. Transcriptional activation of globins and heme-related enzymes starts at the proerythroblast (ProE) stage. Heme and globin cellular concentrations rise from basophilic erythroblast (BasoE) stage to orthochromatic erythroblast (OrthoE) stage, whereas at the same time, several studies demonstrated that mitochondrial content decreases from BasoE stage [5]. Enucleation, the most spectacular event of mammalian erythropoiesis, occurs at the transition from precursor to reticulocyte. During this process, the orthochromatic erythroblast (OrthoE) undergoes enucleation, giving rise to two distinct entities: a pyrenocyte, consisting of the condensed nucleus surrounded by a plasma membrane, and an enucleated reticulocyte that subsequently enters the bloodstream. This process is tightly regulated and requires chromatin condensation, nuclear polarization, actin cytoskeleton remodeling and cytokinesis-like machinery. At the time of enucleation, most of the remaining mitochondria are segregated to the pyrenocyte; however, fully functional mitochondria are still found in newly formed reticulocytes where the final clearance of this organelle occurs [2]. Interestingly mitochondria subcellular localization is an important feature of enucleation and can be easily monitored by transmission electron microscopy. Perinuclear mitochondria and vesicle accumulation are prerequisite to efficient enucleation as mitochondria are thought to provide energy for GTPases-driven membrane constriction, while vesicle accumulation would provide membranes for cytokinesis [6]. Any alteration of mitochondrial clearance would then have a negative outcome on enucleation and contribute to ineffective erythropoiesis. Maintenance of organelles in mature RBCs would also negatively affect the functionality of the cell, and contribute to reducing the RBC lifespan and to anemia. Housekeeping mechanisms encompass proteasome-mediated degradation, micro- and macroautophagy as well as chaperone-mediated autophagy, are tightly connected to vesicular trafficking. While mainly required for daily maintenance to sustain cellular functions of progenitors, these pathways are essential for the profound changes occurring through erythroblasts and reticulocyte’s differentiation.

## 2. Autophagy-Mediated Organelle Clearance During Erythropoiesis

### 2.1. General Description of Autophagy

During the terminal phase of erythroid maturation, cells rely extensively on autophagy, an essential lysosomal degradation pathway responsible for the elimination of cytoplasmic macromolecules and intracellular organelles [7,8]. Originally identified by electron microscopy in the early 1960s, the term autophagy was coined to describe this intracellular “self-eating” process [9]. It is now recognized as the universal mechanism by which eukaryotic cells degrade and recycle their own components through lysosomes, corresponding to vacuolar degradation pathways in plants and yeast.

Autophagy comprises three major pathways, distinguished by the mechanism through which cargo reaches the lysosome: macroautophagy (hereafter referred to as autophagy), microautophagy, and chaperone-mediated autophagy (CMA). During macroautophagy, a cup-shaped membrane, termed the phagophore, elongates and encloses cytoplasmic material within a characteristic double-membrane vesicle known as the autophagosome. Autophagosomes subsequently fuse with endosomes to generate hybrid vesicles called amphisomes, a maturation step for which our group previously identified essential molecular regulators [10]. Ultimately, autophagosomes or amphisomes fuse with lysosomes, where their contents are degraded by lysosomal hydrolases. Studies in yeast and mammalian systems have identified more than 40 autophagy-related (ATG) genes encoding the proteins required for this highly conserved process [11,12].

Macroautophagy can be either non-selective, allowing bulk cytoplasmic degradation during nutrient deprivation, or selective, in which specific adaptor and receptor proteins target defined cargo, including protein aggregates, pathogens, or damaged organelles. By contrast, microautophagy involves direct invagination of the lysosomal membrane to engulf small portions of cytoplasm for degradation [13,14]. In endosomal microautophagy, some intraluminal vesicles formed within multivesicular bodies are secreted as exosomes rather than degraded. Chaperone-mediated autophagy differs fundamentally from both processes, as it does not require membrane remodeling. Instead, soluble cytosolic proteins containing a KFERQ-like motif are recognized by the molecular chaperone HSC70 and directly translocated across the lysosomal membrane through the receptor LAMP2A [15] (Figure 2).

Under nutrient-limiting conditions, autophagy is primarily initiated by inhibition of the mammalian target of rapamycin complex 1 (mTORC1). This releases the inhibitory constraint on the ULK1/ULK2 kinase complex, leading to activation of ULK1 together with its regulatory partners ATG13, ATG101, and FIP200 [16]. Activated ULK1 subsequently stimulates the Beclin 1–VPS34 phosphatidylinositol 3-kinase complex, promoting the local generation of phosphatidylinositol-3-phosphate (PI3P) at endoplasmic reticulum-associated membranes [17,18,19,20]. PI3P recruits WIPI2, which in turn recruits the ATG5–ATG12–ATG16L1 complex, establishing the site for LC3 lipidation [21,22].

LC3 processing is mediated by ATG3 and other ATG proteins, resulting in the conjugation of LC3-I to phosphatidylethanolamine to generate membrane-bound LC3-II [20,23]. LC3-II decorates both the inner and outer membranes of the growing autophagosome, where it promotes phagophore elongation, cargo sequestration, and autophagosome closure [24,25,26]. Selective cargo recognition is mediated by adaptor proteins such as p62/SQSTM1, NDP52, and other LC3-interacting region (LIR)-containing proteins, which bridge ubiquitinated substrates to LC3, thereby ensuring their selective incorporation into autophagosomes [27,28].

Selective degradation of mitochondria, known as mitophagy, represents one of the best-characterized forms of selective autophagy and is essential for mitochondrial quality control. Because dysfunctional mitochondria generate excessive reactive oxygen species (ROS), their efficient removal prevents oxidative damage to proteins, lipids, and nucleic acids, thereby preserving cellular homeostasis [29].

Upon mitochondrial depolarization or structural damage, several mitochondrial outer membrane proteins, including NIX/BNIP3L and FUNDC1, function as ubiquitin-independent mitophagy receptors to recruit the autophagic machinery [30]. The best-characterized canonical pathway involves the PINK1–Parkin axis. Under physiological conditions, PINK1 is continuously imported into healthy mitochondria and rapidly degraded. Loss of mitochondrial membrane potential prevents PINK1 import, resulting in its accumulation on the outer mitochondrial membrane (OMM), where it phosphorylates pre-existing ubiquitin and recruits and activates the E3 ubiquitin ligase Parkin through phosphorylation [31,32,33]. Once active, Parkin generates polyubiquitin chains on OMM proteins that are continuously phosphorylated by PINK1, establishing a feed-forward amplification loop. Phosphorylated ubiquitin chains serve as crucial high-affinity docking sites that essential autophagic adaptors, most prominently optineurin (OPTN) and NDP52, selectively bind to drive localized phagophore nucleation and autophagosomal engulfment [33]. In parallel, activated Parkin ubiquitinates multiple OMM proteins, promoting mitochondrial fragmentation, arresting mitochondrial trafficking, and facilitating the recruitment of secondary receptors such as p62/SQSTM1, which predominantly aids in mitochondrial clustering rather than direct autophagosome recruitment [34]. Together, these coordinated events ensure the selective sequestration of damaged mitochondria within autophagosomes and their subsequent degradation following autophagosome–lysosome fusion.

### 2.2. Mitophagy During Mammalian Erythropoiesis

Terminal erythroid maturation additionally depends on the coordinated activity of several canonical ATG proteins. Transcriptomic analyses have demonstrated that the expression of autophagy-related genes is dynamically regulated during erythropoiesis. Compared with hematopoietic stem and progenitor cells (HSPCs), human basophilic erythroblasts exhibit increased expression of ATG9A, ATG4A, and ATG3, whereas ATG16L1, ATG13, and ATG12 are downregulated [35]. Consistent with these observations, silencing ATG4A in primary human CD34^+^ cells delays erythroid differentiation, demonstrating that autophagy contributes directly to erythroid maturation [35].

Among the core autophagy regulators, ULK1 plays a particularly important role during terminal erythropoiesis. In mice, Ulk1 expression increases during terminal differentiation, coinciding with the elimination of ribosomes and mitochondria [36,37]. Accordingly, Ulk1^−/−^ mice retain both mitochondria and ribosomes in circulating erythrocytes [37]. Surprisingly, mitochondrial membrane potential remains largely preserved in these cells, and treatment with the mitochondrial uncoupler carbonyl cyanide m-chlorophenylhydrazone (CCCP) restores mitochondrial clearance, indicating that alternative mitophagy pathways can compensate for ULK1 deficiency. Likewise, Atg7^−/−^ reticulocytes exhibit only partial impairment of mitochondrial clearance despite developing anemia and reticulocytosis, indicating that canonical ATG5/ATG7-dependent autophagy is important but not absolutely required for organelle elimination [38,39]. Collectively, these findings indicate that both canonical and non-canonical autophagic pathways cooperate during terminal erythroid maturation.

Several non-canonical mechanisms have been proposed to explain ATG5/ATG7-independent mitochondrial clearance. One model suggests that Rab9-dependent autophagosomes generated downstream of ULK1 fuse with late endosomes and Golgi-derived vesicles before lysosomal degradation [40]. Alternatively, damaged mitochondria may be internalized directly into Rab5-positive early endosomes, which subsequently mature into Rab7-positive late endosomes before fusing with lysosomes through an ESCRT-dependent process that bypasses classical autophagosome formation [41,42]. Although these pathways have been demonstrated in other cell types, their contribution to erythroid differentiation remains to be established.

Mitophagy is also tightly controlled at the transcriptional level. During erythropoiesis, the transcription factor NF-E2 promotes mitochondrial clearance by inducing the expression of ULK1 and BNIP3L/NIX [43,44]. Conversely, the KRAB/KAP1-miRNA regulatory network suppresses mitophagy through microRNAs such as miR-351, which targets NIX transcripts [45].

In parallel with autophagy, additional proteolytic pathways may contribute to organelle elimination. 15-Lipoxygenase (15-LOX) has been proposed to permeabilize mitochondrial membranes, thereby facilitating proteasomal degradation during reticulocyte maturation [46]. However, its precise role remains controversial. Alternative studies suggest that 15-LOX promotes mitophagy by disrupting the mitochondrial membrane potential or by oxidizing phospholipids required for LC3 recruitment, although these mechanisms have not been conclusively demonstrated in erythroid cells [47,48]. Clarifying this controversy is crucial for the field, as it defines whether 15-LOX operates through an independent proteasomal pathway or as an upstream lipid-modulating signal that coordinates canonical mitophagy. Unraveling this interplay is essential to determine whether lipid-peroxidation mechanisms can be therapeutically exploited to bypass defective autophagic machinery in erythroid disorders.

Unlike stress-induced mitophagy, mitochondrial clearance during erythroid differentiation primarily targets functionally intact mitochondria. The mitochondrial outer membrane protein BNIP3L/NIX constitutes the principal mitophagy receptor responsible for this physiological process [49,50]. NIX expression increases throughout erythropoiesis, where it promotes mitochondrial depolarization and directly recruits LC3 through its LC3-interacting region (LIR), independently of its BH3 domain [27,51]. Whether NIX-induced depolarization subsequently activates the PINK1/Parkin pathway remains uncertain [52] (Figure 3).

The essential role of NIX has been demonstrated in Nix^−/−^ mice, whose circulating erythrocytes exhibit persistent mitochondrial retention despite apparently normal autophagosome formation [49]. In these animals, failure to undergo the physiological loss of mitochondrial membrane potential prevents efficient mitochondrial sequestration into autophagosomes. Remarkably, treatment with the mitochondrial uncoupler FCCP restores mitochondrial depolarization and rescues mitochondrial engulfment and degradation, indicating that mitochondrial depolarization is a critical step downstream of NIX during erythroid mitophagy.

Finally, several additional mitophagy receptors, including FUNDC1, BCL2L13, OPTN (optineurin), and PHB2 (prohibitin-2), have recently been identified in other physiological settings [53,54,55,56]. Whether these receptors participate in mitochondrial clearance during erythropoiesis remains unknown.

### 2.3. Clearance of Other Organelles

Although mitochondrial elimination has been extensively characterized during terminal erythropoiesis, mature erythrocytes must also eliminate other intracellular organelles, including the endoplasmic reticulum (ER), ribosomes, peroxisomes, lysosomes, and Golgi-derived membranes. Compared with mitophagy, however, the molecular mechanisms governing the clearance of these organelles remain poorly understood.

Current evidence indicates that different organelles are eliminated through partially distinct mechanisms. While BNIP3L/NIX is specifically required for mitochondrial clearance, ULK1 contributes to the elimination of both mitochondria and ribosomes [37,49,50]. Interestingly, Atg7^−/−^ mice exhibit defective mitochondrial clearance but retain the ability to eliminate ribosomes and the ER, suggesting that these organelles can be removed through ATG7-independent autophagic pathways or by alternative degradative mechanisms [57].

Insights from non-erythroid cells support the existence of multiple degradation pathways for other organelles. Peroxisomes can be eliminated through selective autophagy (pexophagy) [58], 15-LOX-mediated degradation [59], or degradation mediated by peroxisomal Lon proteases [60]. Likewise, lysosomal turnover (lysophagy) has been described as a ubiquitin-dependent process requiring the selective autophagy receptor p62/SQSTM1 [61]. Whether similar mechanisms contribute to organelle clearance during erythroid maturation remains unknown.

Following enucleation, reticulocytes undergo a final maturation phase that begins in the bone marrow and is completed in the circulation. During this period, organelle clearance proceeds in parallel with the progressive elimination of residual RNA. Unlike organelle degradation, RNA removal occurs primarily in circulating reticulocytes through ribonuclease-mediated degradation, generating pyrimidine nucleotides that are subsequently hydrolyzed by erythroid pyrimidine 5′-nucleotidase. The physiological importance of this pathway is illustrated by the fact that deficiency of this enzyme causes hereditary hemolytic anemia characterized by pyrimidine nucleotide accumulation within erythrocytes [62].

Transcriptomic analyses have shown that the residual mRNAs present in circulating reticulocytes are predominantly associated with cellular metabolism, membrane transport, and signal transduction [63]. Their gradual elimination is essential for the transition to the metabolically quiescent mature erythrocyte. In parallel, reticulocytes actively remove obsolete membrane proteins through multivesicular bodies and exosome release, highlighting that autophagy, RNA degradation, and exosomal secretion function in concert to complete terminal erythroid maturation.

### 2.4. Membrane Remodeling and Exocytosis

During reticulocyte maturation, plasma membrane remodeling, cell volume reduction, and the selective elimination of unneeded membrane proteins rely on the release of extracellular vesicles (EVs). Importantly, reticulocytes release different types of EVs generated through fundamental, different biogenetic pathways: exosomes and ectosomes (also referred to as microvesicles).

Exosomes are small extracellular vesicles (typically 30–150 nm) generated through the endosomal pathway and secreted by virtually all cell types. Their biogenesis begins with plasma membrane invagination to form early endosomes, which subsequently mature into multivesicular bodies (MVBs, or late endosomes). Fusion of MVBs with the plasma membrane releases their intraluminal vesicles as exosomes into the extracellular space. During reticulocyte maturation, this pathway plays a fundamental role in membrane remodeling, cell volume reduction, and the selective elimination of membrane proteins that are no longer required in mature erythrocytes.

This mechanism was first described in ovine reticulocytes, where the transferrin receptor (TfR/CD71) is internalized through clathrin-mediated endocytosis into 100–200 nm endocytic vesicles before being incorporated into MVBs [64,65]. Rather than undergoing lysosomal degradation, these MVBs fuse with the plasma membrane and release CD71-containing exosomes [66]. The same pathway mediates the removal of several proteins that are essential during erythropoiesis but absent from mature red blood cells, including the glucose transporter GLUT1, acetylcholinesterase, α4β1 integrin, and aquaporin-1 [65,67,68,69]. In contrast, major cytoskeletal proteins such as spectrin and actin are excluded from these vesicles and remain associated with the mature erythrocyte membrane [70].

In contrast, ectosomes are larger vesicles (typically 100–1000 nm) produced directly through outward budding and shedding of the plasma membrane. This process involves local cytoskeletal reorganization, flippase/floppase-mediated lipid asymmetry changes, and specialized plasma membrane microdomains enriched in sphingomyelin and cholesterol that facilitate membrane protrusion and vesicle release. To maintain rigor, the manuscript avoids using the term "exosome" as a generic synonym for all extracellular vesicles shed by reticulocytes, reserving it strictly for vesicles whose endosomal/MVB origin is established, while referring to plasma membrane-derived vesicles as EVs.

Increasing evidence indicates that exosome biogenesis and autophagy are closely interconnected during terminal erythroid maturation. Although autophagy is indispensable for organelle clearance, the final stages of reticulocyte maturation are characterized by progressive disappearance of the lysosomal compartment, while lysosomal proteins such as LAMP2 are themselves eliminated through exocytosis [71]. These observations suggest that degradation of residual organelles cannot rely exclusively on classical lysosomal proteolysis.

Supporting this concept, LC3 has been detected on the membranes of glycophorin A (GPA)-positive endosomes, suggesting the formation of hybrid autophagic-endosomal compartments that contain mitochondria, Golgi membranes, and lysosomal components [72]. These structures may arise through fusion between autophagosomes and endocytic vesicles before their extracellular release, thereby coupling autophagy with exosome-mediated disposal of residual intracellular material. Although the molecular mechanisms governing this crosstalk remain incompletely understood, this pathway likely represents a key mechanism for completing reticulocyte maturation after the loss of functional lysosomes.

The spleen also appears to contribute to this remodeling process. Splenectomized individuals accumulate large intracellular vacuoles within circulating reticulocytes, indicating impaired clearance of residual vesicular structures [73]. In addition, specialized plasma membrane domains enriched in sphingomyelin and cholesterol facilitate membrane budding and vesicle release, highlighting the importance of membrane lipid organization during EVs biogenesis [74].

## 3. Autophagy Defects in Hematological Diseases

Before examining individual erythroid disorders, a fundamental conceptual framework must be established regarding the origin of mitochondrial retention: distinguishing primary defects of the autophagic machinery from secondary consequences of mitochondrial dysfunction. On one hand, primary mitophagy defects stem from intrinsic impairments in targeted degradation pathways (e.g., loss or downregulation of NIX or ULK1), which directly block organelle clearance during terminal reticulocyte maturation. On the other hand, primary metabolic, transcriptional, or structural perturbations, such as impaired heme biosynthesis, respiratory chain defects, or microenvironmental inflammatory stress, primarily drive mitochondrial dysfunction, iron overload, and ROS generation. Under these conditions, secondary alterations or insufficient capacity of the autophagic flux fail to cope with organelle damage, ultimately accelerating cell death and ineffective erythropoiesis. Framing erythroid pathologies along this primary or secondary defect is critical for understanding their molecular etiology and identifying stage-specific therapeutic interventions.

### 3.1. Sickle Cell Disease (SCD)

Sickle cell disease (SCD) is a highly prevalent hemoglobinopathy characterized by chronic hemolysis, recurrent vaso-occlusive episodes, and persistent inflammation. Despite extensive research into the molecular and cellular mechanisms underlying SCD, several aspects of its pathophysiology remain incompletely understood.

A few years ago, a novel population of circulating red blood cells (RBCs) retaining mitochondria was identified in patients with SCD [75]. Since then, several independent studies have confirmed that a subset of SCD patients exhibits an abnormally high proportion of mature erythroid cells retaining mitochondria [75,76,77,78,79,80]. Mitochondrial retention has been observed not only in reticulocytes but also in mature circulating RBCs, indicating a defect in mitochondrial clearance during terminal erythropoiesis [77,80].

Importantly, these retained mitochondria remain metabolically active, displaying functional respiratory chain complexes and tricarboxylic acid (TCA) cycle enzymes, resulting in increased reactive oxygen species (ROS) production [75,76,78]. Consequently, patients with mitochondria-retaining RBCs exhibit several pathological features, including lower hematocrit, increased phosphatidylserine exposure, reduced deformability, elevated bilirubin and lactate dehydrogenase levels, and lower fetal hemoglobin (HbF) expression, all of which are associated with enhanced hemolysis [76,77,79,80].

Although several independent research groups have consistently demonstrated the presence of mitochondria-retaining erythroid cells in SCD, direct comparison between published studies remains challenging. This is largely due to incomplete reporting of key methodological details and patient characteristics, including the approaches used to identify mitochondria-retaining cells and the clinical status of the patients analyzed. The number of patients or samples examined also varies considerably, ranging from 6 patients in [75] to 101 in [78]. Transfusion status was reported in some studies [76,77,80], but not in others [75,78,79]. Similarly, hydroxyurea (HU) treatment was reported in some cohorts [77,79,80], with either no significant effect on mitochondrial retention [77,80] or a slight reduction [79].

The potential contribution of contaminating or immature blood cells should also be considered. The presence of residual platelets or white blood cells is not systematically reported, although in at least one study these populations represented less than 0.5% of the analyzed cells [76]. Importantly, most studies identify and analyze mature RBCs and reticulocytes using specific flow-cytometry gating strategies. Nevertheless, differences in gating criteria may introduce an additional source of variability when comparing mitochondrial retention across studies.

This methodological heterogeneity is particularly relevant to the unresolved question of the functional state of retained mitochondria. Based on measurements of mitochondrial membrane potential (ΔΨm) and ROS production in gated mature RBCs and reticulocytes, Martino et al. reported that retained mitochondria were functionally active and produced ROS in reticulocytes, whereas mitochondrial activity was not detected in mature RBCs [77]. Moriconi et al. performed metabolic functional assays on samples from three patients and demonstrated oxidative phosphorylation (OXPHOS) activity; however, the study did not indicate whether residual reticulocytes, platelets, or white blood cells were present in the analyzed samples [78]. Similarly, Esperti et al. measured oxygen consumption in mitochondria-containing RBCs and demonstrated respiratory activity that was sensitive to respiratory-chain inhibitors [76].

These apparently divergent observations regarding mitochondrial respiratory activity may, at least in part, reflect differences in the cellular populations included in the analyses. In particular, the presence of immature reticulocytes within populations classified as mature RBCs could contribute to the detection of OXPHOS activity. Although most studies define mature RBCs as GPA^+^/CD71^−^ cells, Romana et al. demonstrated that peripheral blood from patients with SCD contains a significant population of GPA^+^/CD71^−^/TO^+^ reticulocytes and proposed that only GPA^+^/CD71^−^/TO^−^ cells should be considered fully mature RBCs [77]. These GPA^+^/CD71^−^/TO^+^ cells may therefore contribute to the detection of active mitochondrial respiration in apparently mature RBC populations, consistent with the observation by Martino et al. that mitochondrial activity and ROS production are detectable in reticulocytes but not in mature RBCs [77].

Taken together, these observations highlight the importance of standardized definitions and gating strategies for distinguishing mature RBCs from mitochondria-retaining reticulocytes. Harmonization of patient characterization, transfusion and treatment status, mitochondrial detection methods, and flow-cytometry gating will be essential to determine whether retained mitochondria remain functionally active throughout RBC maturation or progressively lose their respiratory capacity before or after entry into the circulation.

The molecular mechanisms responsible for mitochondrial persistence remain poorly understood. Martino et al. (2021) reported that reduced expression of the mitophagy regulators NIX and PINK1 correlates with mitochondrial retention in circulating RBCs, supporting the hypothesis that defective mitophagy contributes to this phenotype [77]. More recently, Romana et al. demonstrated that both HbSS and HbSC patients display increased proportions of mature RBCs retaining mitochondria [80]. Although mitochondrial retention strongly correlated with markers of hemolysis, no association with systemic inflammatory markers was observed. As expected, HbSC patients exhibited an intermediate phenotype between healthy individuals and HbSS patients.

Beyond their contribution to oxidative stress, retained mitochondria may actively participate in the chronic inflammatory state characteristic of SCD. Metabolically active mitochondria can stimulate innate immune responses by promoting neutrophil production of type I interferons, thereby amplifying inflammation [78].

These observations also highlight mitochondrial clearance as a potential therapeutic target. Pharmacological activation of autophagy using the mTORC1 inhibitor sirolimus significantly reduced mitochondrial retention and oxidative stress in the Townes SCD mouse model [75], supporting the concept that restoration of mitophagy may improve erythroid maturation. Similarly, mitapivat, a pyruvate kinase activator currently under clinical development for SCD, has also been shown to decrease mitochondrial retention in the Townes mouse model [81].

Together, these preclinical and observational findings identify defective mitochondrial clearance as a previously underappreciated feature of SCD pathophysiology and support further investigation of mitophagy-modulating approaches as potential complementary therapeutic strategies to reduce hemolysis, oxidative stress, and inflammation in patients with SCD.

### 3.2. β-Thalassemia

In contrast to sickle cell disease, β-thalassemia is characterized by the accumulation of excess α-globin chains in erythroblasts. These unstable α-globin chains form cytotoxic intracellular precipitates that impair erythroid maturation, ultimately causing severe anemia (reviewed in [82]). In addition, heme released from denatured α-globin can further increase oxidative and proteotoxic stress. Surprisingly, despite this hostile intracellular environment, there is no evidence of a massive increase in apoptosis in β-thalassemic erythroblasts, suggesting that compensatory cytoprotective mechanisms are activated.

Several studies indicate that autophagy is one of these protective mechanisms. Increased autophagic activity has been consistently observed during erythropoiesis of β-thalassemic erythroblasts, both in vitro and in vivo [83,84]. In cultured CD34^+^ cells from β-thalassemia patients, Wu and colleagues demonstrated enhanced mitophagy, characterized by increased mitochondria-lysosome colocalization, elevated LC3-II and BNIP3L/NIX expression, and degradation of p62, indicating activation of selective mitochondrial clearance [85]. Likewise, increased numbers of autophagosomes and enhanced LC3-LAMP1 colocalization have been reported in bone marrow erythroblasts from both β-thalassemic mice and β^0^/HbE patients [86].

Compelling evidence demonstrates that autophagy directly contributes to the degradation of excess α-globin. Lechauve and colleagues showed that deletion of the autophagy-activating kinase ULK1 markedly reduces autophagic clearance of insoluble α-globin in β-thalassemic erythroblasts, aggravating ineffective erythropoiesis and anemia. In contrast, deletion of the canonical autophagy gene Atg5 had only minor effects, revealing the existence of a non-canonical ULK1-dependent, ATG5-independent autophagic pathway [84]. These finding are consistent with the ATG5-independent mechanisms previously described by Honda et al. in reticulocytes from Atg5^−/−^ mice [87]. This pathway is also LC3-independent and involves direct Rab9-dependent fusion of phagophores with late endosomes or trans-Golgi-derived vesicles [88].

Consistent with these findings, pharmacological inhibition of mTORC1 by rapamycin stimulated ULK1-dependent autophagy, reduced α-globin precipitation, and significantly improved erythropoiesis, hemoglobin levels, reticulocyte counts, and splenomegaly in β-thalassemic mice. Similar reductions in free α-globin accumulation were also observed in erythroblasts differentiated from CD34^+^ cells of β-thalassemia patients [84].

The importance of the ULK1 pathway has subsequently been reinforced by several independent studies. Disruption of the bicistronic microRNA locus miR-144/451 alleviated β-thalassemia by reducing mTORC1 activity and stimulating ULK1-mediated autophagic degradation of free α-globin in both Hbb^th3/- mice and patient-derived CD34^+^ erythroblasts [89]. Furthermore, rapamycin and the dual mTORC1/mTORC2 inhibitor AZD8055 both improved erythroid maturation and anemia in murine models of β-thalassemia, although AZD8055 produced a stronger activation of autophagy [90]. Supporting the translational relevance of these observations, erythroid precursor cells isolated from β-thalassemia patients treated with low-dose sirolimus displayed a ten-fold increase in ULK1 expression, consistent with results obtained in patients enrolled in the Sirthalaclin pilot clinical trial (NCT03877809) [91,92,93].

Additional evidence indicates that autophagy protects β-thalassemic erythroblasts from cellular stress. Pharmacological inhibition of autophagy with chloroquine significantly increased erythroblast apoptosis and phosphatidylserine exposure, demonstrating that autophagy limits cell death during ineffective erythropoiesis [86]. Likewise, inhibition of autophagic flux impaired α-globin degradation to an extent comparable to proteasome inhibition, whereas simultaneous inhibition of both pathways produced additive effects, highlighting the complementary roles of autophagy and the ubiquitin-proteasome system in maintaining erythroid proteostasis [94].

Despite this increased autophagic activity, organelle quality control appears to remain defective. Suriyun and colleagues reported delayed terminal erythroid maturation and impaired mitochondrial clearance in β^0^/HbE erythroblasts, even though the mitophagy regulators BNIP3L/NIX and PINK1 were overexpressed [95]. These findings suggest that activation of mitophagy signaling is insufficient to ensure efficient organelle elimination, pointing toward defects in downstream regulatory mechanisms controlling autophagic flux.

Autophagy dysregulation also extends beyond terminal erythropoiesis. β-Thalassemic erythroblasts exhibit an altered unfolded protein response (UPR), remaining responsive to intracellular stress induced by tunicamycin but showing defective responses to extracellular stress such as cytokine deprivation [83]. More recently, single-cell RNA sequencing revealed altered TGF-β signaling and autophagy transcriptional signatures in hematopoietic stem and multipotent progenitor cells from β-thalassemia patients. Reduced autophagic activity was associated with loss of stem cell dormancy, whereas pharmacological stimulation of autophagy restored hematopoietic homeostasis, identifying the interplay between TGF-β signaling and autophagy as an important regulator of ineffective erythropoiesis [96].

Collectively, these studies establish autophagy as a major cytoprotective mechanism in β-thalassemia that limits α-globin toxicity, preserves erythroid cell survival, and ameliorates ineffective erythropoiesis. Nevertheless, despite increased autophagic activity, mitochondrial clearance and terminal erythroid maturation remain defective, indicating that critical regulatory pathways controlling autophagic flux are still poorly understood. Better understanding how autophagy is regulated during terminal erythropoiesis—including the roles of the PI3K/Akt/mTOR pathway, ULK1 signaling, and autophagy-regulating microRNAs—may reveal novel biomarkers and therapeutic targets for β-thalassemia. In this context, identifying the molecular mechanisms coordinating autophagy with organelle clearance during erythroid maturation represents an important unmet challenge.

### 3.3. Myelodysplastic Syndromes (MDS)

Ineffective erythropoiesis is a defining feature of myelodysplastic syndromes (MDS), a heterogeneous group of clonal hematopoietic stem cell disorders characterized by impaired erythroid maturation and bone marrow failure. Increasing evidence indicates that mitochondrial dysfunction and defective autophagic clearance are major contributors to MDS pathophysiology [97,98]. During normal terminal erythropoiesis, organelle elimination and exosomal shedding of obsolete membrane proteins are tightly coordinated to generate mature erythrocytes. In MDS, this highly regulated remodeling process is disrupted at multiple stages of erythroid differentiation.

In high-risk MDS, NIX-dependent mitophagy is markedly impaired in bone marrow nucleated red blood cells (NRBCs) [99]. This defect is characterized by reduced expression of the mitophagy receptor NIX (BNIP3L) and the autophagosomal marker LC3-II, together with decreased autophagosome formation [99,100]. Mechanistically, impaired mitophagy is associated with downregulation of the autophagy regulators AMPK and ULK1, accompanied by aberrant activation of the autophagy inhibitor mTOR [99]. As a consequence, dysfunctional mitochondria accumulate within erythroid precursors, as reflected by increased expression of the mitochondrial outer membrane protein TOM20. Mitochondrial retention is accompanied by loss of mitochondrial membrane potential (ΔΨm), excessive production of reactive oxygen species (ROS), and enhanced intramedullary apoptosis, thereby directly contributing to ineffective erythropoiesis.

As erythroblasts mature into reticulocytes, mitochondrial clearance must be coordinated with the exosomal elimination of the transferrin receptor CD71. In a subset of MDS patients, these two processes become uncoupled [101]. Mitochondrial abnormalities, or deficiency of the immediate early response gene IEX-1, promote premature mitochondrial elimination. The resulting decrease in intracellular ROS may impair multivesicular body (MVB)-mediated exosome release, leading to delayed CD71 shedding. Consequently, exosomal shedding of CD71 is delayed, leading to the accumulation of an abnormal population of CD71-positive/mitochondria-negative (CD71^+^/Mito^−^) reticulocytes [101]. Because CD71-containing exosomes also contribute to cellular iron disposal, defective exosome release may further impair iron homeostasis. Clinically, the proportion of these abnormal reticulocytes correlates with the degree of mitochondrial membrane depolarization observed in MDS patients.

The importance of mitochondrial quality control is particularly evident in MDS with ring sideroblasts (MDS-RS), a subtype frequently associated with somatic mutations in the RNA splicing factor SF3B1 [97]. These mutations alter the splicing of several genes involved in mitochondrial heme synthesis and iron metabolism, including PPOX, TMEM14C, and ABCB7, thereby impairing mitochondrial iron utilization. As a result, iron accumulates within mitochondria, giving rise to the characteristic perinuclear ring sideroblasts. The abundance of these iron-loaded mitochondria correlates with both ring sideroblast frequency and the severity of anemia [99].

Beyond intrinsic erythroid defects, the bone marrow microenvironment also contributes to ineffective erythropoiesis in MDS [97]. Bone marrow endothelial progenitor cells (BM-EPCs) from MDS patients display increased ROS production, enhanced basal apoptosis, and progressive functional impairment that parallels disease severity [100]. In co-culture experiments, these dysfunctional EPCs fail to support normal hematopoietic stem cells and instead promote ROS accumulation and apoptosis in healthy progenitors, thereby aggravating ineffective hematopoiesis [100]. In parallel, hyperactivation of the TGF-β/SMAD signaling pathway further amplifies erythroid failure by altering mitochondrial function, promoting mitochondrial depolarization, and reinforcing the pro-apoptotic bone marrow environment [97,98]. Consequently, therapeutic approaches aimed at restoring mitophagic flux or inhibiting aberrant TGF-β signaling, including the use of TGF-β ligand traps such as luspatercept, have emerged as promising strategies to improve erythropoiesis in MDS [98].

### 3.4. Fanconi Anemia (FA)

Fanconi anemia (FA) is an inherited bone marrow failure syndrome characterized by chromosomal instability, progressive pancytopenia, congenital abnormalities, and a markedly increased risk of hematological malignancies and solid tumors [102,103]. The disease results from pathogenic variants in one of more than 20 genes encoding components of the Fanconi anemia (FANC) DNA repair pathway, which coordinates the repair of DNA interstrand crosslinks (ICLs) through activation of the FANCD2/FANCI complex and the downstream homologous recombination machinery [104]. Although defective DNA repair has long been considered the primary pathogenic mechanism underlying FA, accumulating evidence indicates that several FANC proteins also perform essential cytoplasmic functions involved in mitochondrial homeostasis, oxidative metabolism, and selective autophagy [105,106].

Among these non-canonical functions, FANCC was the first FANC protein shown to participate directly in selective autophagy by promoting the clearance of damaged mitochondria independently of its role in DNA repair [105]. More recently, FANCD2 and FANCL have also been identified as key regulators of mitophagy and mitochondrial dynamics. Interestingly, FANCL-dependent mitophagy appears to occur largely independently of its E3 ubiquitin ligase activity, suggesting that FANCL may act primarily as a structural scaffold that facilitates recruitment or stabilization of the mitophagy machinery rather than functioning exclusively through substrate ubiquitination [106]. These findings substantially broaden the biological functions of the FANC pathway, establishing a direct connection between genome maintenance and mitochondrial quality control.

Consistent with these observations, cells derived from FA patients exhibit profound mitochondrial abnormalities, including impaired oxidative phosphorylation, altered mitochondrial dynamics, and increased production of reactive oxygen species (ROS), indicating that mitochondrial dysfunction represents a major pathogenic mechanism in addition to defective DNA repair [102,107]. The persistence of dysfunctional mitochondria resulting from defective mitophagy further amplifies oxidative stress, leading to oxidative DNA damage, activation of p53-dependent stress responses, and progressive exhaustion of hematopoietic stem cells. These events ultimately impair hematopoietic homeostasis and compromise erythroid differentiation [102,103].

The identification of FANCC, FANCD2, and FANCL as regulators of mitochondrial quality control provides an important mechanistic framework linking defective mitophagy to the hematopoietic manifestations of FA [105,106]. Although the precise contribution of impaired mitophagy to ineffective erythropoiesis remains to be fully elucidated, accumulating evidence supports a model in which defective mitochondrial clearance sustains oxidative stress, metabolic dysfunction, and hematopoietic stem cell attrition, thereby contributing to progressive bone marrow failure.

Collectively, these findings demonstrate that the pathophysiology of Fanconi anemia extends well beyond defective DNA repair. The emerging role of FANC proteins in selective autophagy and mitochondrial quality control establishes a functional link between genome stability, mitochondrial homeostasis, and erythropoiesis, highlighting these pathways as promising therapeutic targets for preserving hematopoietic stem cell function and improving erythroid output.

### 3.5. Diamond–Blackfan Anemia (DBA) 

Diamond–Blackfan anemia (DBA) is a congenital erythroid aplasia caused predominantly by heterozygous mutations in genes encoding ribosomal proteins, including RPS19, RPL5, RPL11, and RPS26, among other ribosomal components [108,109]. These mutations impair ribosome biogenesis and induce ribosomal stress, leading to TP53 stabilization, cell-cycle arrest, and apoptosis of erythroid progenitors, ultimately resulting in ineffective erythropoiesis [109,110]. Although ribosomal dysfunction remains the defining feature of DBA, accumulating evidence suggests that additional processes, including translational dysregulation, altered cellular homeostasis, inflammation, and autophagy, may contribute to the selective vulnerability of the erythroid lineage [108,111].

Using hematopoietic progenitors differentiated from patient-derived iPSCs, Doulatov and colleagues recapitulated the characteristic erythroid differentiation defect and performed a high-throughput chemical screen to identify compounds capable of restoring erythropoiesis [112]. This approach identified SMER28, a small molecule previously described as an mTOR-independent inducer of autophagy. Mechanistic studies showed that SMER28 enhanced autophagic flux and significantly improved erythroid differentiation through an ATG5-dependent mechanism [112]. Importantly, genetic suppression of ATG5 abolished the beneficial effect of SMER28, providing functional evidence that autophagy is required for the erythroid rescue observed in this experimental model [111].

These findings should nevertheless be interpreted with caution. Rather than demonstrating that DBA is primarily caused by defective autophagy, they indicate that pharmacological activation of autophagy can partially restore erythroid maturation despite the underlying ribosomal defect. Recent reviews have incorporated these observations into the broader molecular framework of DBA and suggest that modulation of autophagy may influence erythroid homeostasis alongside p53 activation, translational defects, inflammation, and alterations in heme and globin synthesis. However, direct evidence supporting impaired autophagy as a primary pathogenic mechanism remains limited, and the molecular links between ribosomal stress and autophagy have yet to be fully defined [108,111].

Taken together, current evidence supports the view that autophagy represents a potential therapeutic target rather than an established primary disease mechanism in Diamond–Blackfan anemia. Further investigation into the interplay between ribosomal stress, proteostasis, metabolic adaptation, and autophagy will be essential to determine how these pathways cooperate during erythropoiesis and whether pharmacological modulation of autophagy can ultimately be translated into effective therapeutic strategies.

### 3.6. X-Linked Sideroblastic Anemia (XLSA)

X-linked sideroblastic anemia (XLSA) provides a relevant model to examine how defective heme biosynthesis intersects with mitochondrial iron retention, oxidative injury, ferroptotic vulnerability, and autophagy-dependent erythroid remodeling. Classical XLSA is caused by germline loss-of-function mutations in ALAS2, which encodes the erythroid-specific 5-aminolevulinate synthase, the first and rate-limiting enzyme of heme biosynthesis [113].

During normal erythroid differentiation, mitochondria have a temporally restricted dual function: they are first required for intensive heme production, but must subsequently be removed to allow terminal maturation into mitochondria-free red cells. This transition is partly coordinated by GATA1, a master erythroid transcription factor that activates autophagy-related gene programs, directly and through FOXO3, including genes involved in autophagosome formation and mitochondrial clearance such as MAP1LC3B, GABARAP, and BNIP3L/NIX [114]. In addition, heme itself has been shown to promote the expression of GATA1-regulated mitophagy-related genes and to induce mitophagy during murine erythroid maturation, supporting a model in which mitochondria first generate heme and heme then contributes to the transcriptional program that promotes mitochondrial clearance [115]. XLSA disrupts this coordination at its metabolic origin.

In HUDEP-2 erythroid models carrying ALAS2 R170L or ALAS2 R170H mutations, defective heme synthesis causes impaired hemoglobinization, reduced terminal erythroid maturation, ring sideroblast formation, abnormal mitochondrial iron deposits, mitochondrial spheroids, increased labile iron, lipid peroxidation, and enhanced susceptibility to ferroptosis. Gene Ontology enrichment analysis of the upregulated gene set identified autophagy as one of the most significantly enriched biological processes [116]. Mechanistically, heme deficiency stabilizes the heme-sensitive transcriptional repressor BACH1, which represses genes involved in glutathione synthesis and iron homeostasis, including GCLM, GCLC, FTH1, FTL, and SLC40A1, thereby reducing antioxidant capacity and increasing vulnerability to iron-dependent lipid damage [117]. A subsequent study further refined this model by showing that ALAS2-mutant XLSA clones accumulate GATA1 protein but display impaired expression of GATA1-dependent autophagy-related genes, including BNIP3L, MAP1LC3B, and GABARAP, together with reduced autophagic activity, accumulation of large autophagolysosomes, altered GATA1-associated protein complexes, suppression of NCOA4, and increased ferritin protein accumulation, consistent with defective ferritinophagy [116].

This creates a paradoxical situation: XLSA erythroblasts are biochemically primed for ferroptosis through iron overload, glutathione depletion, and lipid peroxidation, yet impaired autophagy/ferritinophagy may limit the release of ferritin-bound iron and thereby prevent full execution of ferroptotic death unless an additional inducer, such as erastin, is applied. Thus, XLSA should be placed in this field as a disease of mitochondrial iron mismanagement with potential secondary consequences on autophagy-dependent mitochondrial quality control, rather than as a proven primary mitophagy disorder. This distinction is important: the best-demonstrated abnormality is mitochondrial iron retention caused by ALAS2-dependent heme deficiency, whereas true mitochondrial organelle retention due to defective mitophagic clearance remains a plausible but incompletely demonstrated mechanism. Nevertheless, because heme promotes the mitophagy program and GATA1 coordinates autophagy-related erythroid maturation, impaired heme production in XLSA may weaken the normal coupling between mitochondrial function, ferritin turnover, ferroptosis control, and mitochondrial removal.

XLSA therefore occupies an important position within the broader theme of autophagy defects and mitochondrial retention in erythroid diseases: it links defective heme synthesis, iron-loaded mitochondria, altered GATA1-dependent autophagy, impaired ferritinophagy, oxidative stress, ferroptotic susceptibility, and potentially insufficient autophagy/mitophagy activation during terminal erythropoiesis.

### 3.7. Pearson Syndrome

Pearson syndrome (PS) is a rare and often fatal mitochondrial disorder caused by large-scale deletions of mitochondrial DNA (mtDNA). It typically presents in infancy with sideroblastic anemia and progressive multisystem complications resulting from impaired oxidative phosphorylation (OXPHOS) [118,119,120]. Hematological manifestations include macrocytic sideroblastic anemia associated with bone marrow failure, neutropenia, thrombocytopenia, elevated fetal hemoglobin, and characteristic bone marrow dysplasia, including cytoplasmic vacuolization of erythroid and myeloid precursors. Ring sideroblasts are observed in approximately 70–85% of patients and reflect the accumulation of iron-loaded mitochondria within erythroid precursors [119,120].

At the molecular level, large-scale mtDNA deletions remove genes encoding essential subunits of the mitochondrial respiratory chain. These deletions result in severe respiratory-chain dysfunction, including cytochrome c oxidase (COX) deficiency, impaired ATP production, and profound alterations in mitochondrial metabolism in hematopoietic cells [118,120,121].

Although defective mitochondrial clearance has been proposed as a potential contributor to the erythroid phenotype of Pearson syndrome, direct evidence supporting this hypothesis remains lacking. In principle, mitochondria carrying large-scale mtDNA deletions represent damaged organelles that would be expected to be recognized by mitochondrial quality-control pathways. However, to date, no study has directly quantified autophagic or mitophagic flux in erythroid cells derived from patients with Pearson syndrome.

Several observations nevertheless support a potential link between mitochondrial dysfunction and defective mitochondrial elimination [122]. Large-scale mtDNA deletions result in the loss of mtDNA-encoded respiratory-chain subunits, leading to severe OXPHOS deficiency and reduced COX activity in erythroid precursors [118,120,121]. Impaired respiratory-chain function can disrupt mitochondrial iron metabolism by affecting heme synthesis and iron-sulfur cluster biogenesis, thereby promoting intramitochondrial iron accumulation and contributing to the formation of characteristic ring sideroblasts. This mechanism is consistent with the broader pathophysiology of congenital sideroblastic anemias, in which defects affecting mitochondrial heme synthesis, iron-sulfur cluster assembly, or mitochondrial protein translation converge on mitochondrial iron accumulation and ineffective erythropoiesis [123,124].

Indirect evidence also suggests that mitochondrial dysfunction associated with mtDNA deletions can activate cellular quality-control pathways. Alemi et al. (2007) reported that mtDNA deletions inhibit proteasomal activity while increasing the expression of autophagy-related transcripts, including ATG12, suggesting activation of an autophagic response to mitochondrial stress [125]. However, transcriptional induction of autophagy-related genes does not necessarily indicate effective autophagic flux or successful mitochondrial degradation. Thus, it remains unknown whether the activation of autophagy in Pearson syndrome results in efficient elimination of mitochondria carrying mtDNA deletions.

The persistence of mitochondria in mature erythrocytes has been reported in patients with mitochondrial disorders, including Pearson syndrome, raising the possibility that mitochondrial clearance during terminal erythropoiesis may be incomplete [122]. Such mitochondrial retention could potentially increase oxidative stress, promote cellular damage, and contribute to reduced erythrocyte survival and ineffective erythropoiesis. However, these mechanisms remain hypothetical, as mitochondrial clearance and mitophagic flux have not yet been directly quantified in primary erythroid cells derived from patients with Pearson syndrome using contemporary approaches that distinguish mitochondrial content from effective mitophagic degradation.

Collectively, current evidence supports a model in which large-scale mtDNA deletions drive respiratory-chain dysfunction, impaired iron utilization, oxidative stress, and ineffective erythropoiesis, contributing to the sideroblastic phenotype of Pearson syndrome. Whether defective mitochondrial clearance further contributes to this pathogenesis remains unresolved, and clarifying this point could identify mitochondrial quality-control pathways as therapeutic targets to improve erythroid maturation.

### 3.8. Aplastic Anemia

Aplastic anemia (AA) is a bone marrow failure syndrome characterized by hypocellular bone marrow and peripheral pancytopenia resulting from impaired hematopoiesis [126]. Bone marrow failure may affect one or more hematopoietic lineages and encompasses a heterogeneous group of disorders. According to their etiology, bone marrow failure syndromes can be broadly classified as iatrogenic, inherited, or immune-mediated [127]. Iatrogenic forms result from damage to the hematopoietic stem and progenitor cell (HSPC) compartment following exposure to external agents, including drugs, radiation, or toxic chemicals. Inherited bone marrow failure syndromes are caused by germline mutations affecting HSPCs and other pathways essential for genome maintenance, telomere biology, or cellular homeostasis. In contrast, acquired aplastic anemia is primarily an immune-mediated disorder in which HSPCs are targeted by an aberrant autoimmune response [128].

Immune-mediated destruction of HSPCs is the central pathogenic mechanism underlying acquired AA [128]. This process leads to bone marrow hypocellularity and peripheral pancytopenia through a combination of direct cytotoxicity and cytokine-mediated suppression of hematopoiesis. Cytotoxic T lymphocytes (CTLs) play a central role by eliminating HSPCs through complementary mechanisms [129]. Activated CTLs release perforin and granzyme B, inducing apoptosis in target HSPCs. Perforin forms pores in the target-cell membrane, allowing granzyme B to enter the cytoplasm and activate caspase-dependent apoptotic pathways [128]. In addition, HSPCs from patients with AA exhibit increased expression of the Fas receptor, rendering them more susceptible to Fas ligand (FasL)-induced apoptosis. FasL binding to Fas activates the extrinsic apoptotic pathway and further contributes to HSPC depletion [130].

The pathogenic immune response is also sustained by inflammatory cytokines. Activated CTLs and T helper 1 (Th1) cells produce high levels of interferon-γ (IFN-γ) and tumor necrosis factor-α (TNF-α), which contribute substantially to disease progression [128]. IFN-γ enhances Fas-mediated apoptosis by inducing Fas expression through the interferon regulatory factor-1 (IRF-1) pathway, thereby increasing HSPC sensitivity to apoptotic stimuli. IFN-γ can also directly impair hematopoiesis by interfering with the differentiation of multipotent progenitors (MPPs) toward common myeloid progenitors (CMPs), thereby affecting the production of granulocytes, monocytes, erythrocytes, and platelets [129]. In addition, IFN-γ has been reported to interfere with thrombopoietin signaling and to promote the expression of interleukin-15 (IL-15), which may further inhibit HSPC proliferation [128,131].

TNF-α is another important inflammatory mediator in AA and is frequently elevated in affected patients. Through activation of TNF receptor 1 (TNFR1), TNF-α can suppress HSPC proliferation and promote apoptotic signaling, including through the induction of Fas ligand and other pro-apoptotic pathways [130]. Together, these mechanisms establish a self-reinforcing cycle of immune-mediated HSPC destruction and impaired hematopoiesis.

The persistent inflammatory environment in AA is also associated with increased oxidative stress and metabolic alterations within the bone marrow niche. Exposure of CD34^+^CD38^−^ HSPCs and CD34^+^CD38^+^ progenitors to IFN-γ and TNF-α induces the expression of inducible nitric oxide synthase (iNOS), leading to increased nitric oxide (NO) production [130]. NO can act as a paracrine cytotoxic mediator, contributing to the damage of neighboring hematopoietic cells. Increased NO production may promote oxidative and nitrosative stress, DNA damage, and mitochondrial dysfunction, thereby further compromising HSPC survival and function.

In HSPCs, basal autophagy is essential for maintaining cellular quiescence, self-renewal, and long-term regenerative potential, whereas selective mitophagy contributes to mitochondrial quality control and limits the accumulation of dysfunctional mitochondria and excessive reactive oxygen species (ROS) [132]. These observations raise the possibility that alterations in autophagy may contribute to HSPC dysfunction in AA.

However, in contrast to the well-established role of immune-mediated HSPC destruction, the contribution of defective autophagy to AA pathogenesis remains incompletely defined. Chronic exposure to inflammatory cytokines such as IFN-γ and TNF-α may impose sustained oxidative and metabolic stress on HSPCs, conditions that would normally activate autophagy as an adaptive response. Prolonged or excessive stress, however, may overwhelm these protective mechanisms, resulting in impaired mitochondrial quality control, increased ROS accumulation, metabolic dysfunction, and reduced stem cell fitness. Thus, defective autophagy and mitophagy may represent secondary mechanisms that exacerbate HSPC dysfunction rather than primary drivers of AA. Notably, direct evidence for mitochondrial retention in AA has not been reported to date, indicating that its relevance to aplastic anemia remains unresolved and warrants further investigation.

Recent studies have identified extracellular vesicle-associated microRNAs as potential biomarkers and regulators of HSPC biology in AA. Among these, miR-126-5p has emerged as a potential diagnostic and prognostic biomarker [128], regulating several processes relevant to HSPC homeostasis, including quiescence, glucose metabolism, and autophagy, in part through modulation of PI3K/AKT/mTOR signaling. This axis is a central regulator of nutrient sensing and energy metabolism: mTOR inhibition induces autophagy, promoting the recycling of damaged cellular components and adaptation to metabolic stress. Altered miR-126-5p expression in AA could therefore influence this signaling network and potentially modify autophagic activity in HSPCs. Nevertheless, whether these alterations directly impair autophagic flux in AA HSPCs remains to be established.

The potential involvement of autophagy in AA is particularly relevant to erythropoiesis. As a consequence of chronic immune-mediated HSPC injury, the bone marrow of patients with AA exhibits a profound reduction in the CD34^+^ stem and progenitor cell compartment and persistent dysfunction of residual progenitors. IFN-γ can induce intrinsic abnormalities that impair progenitor responses to differentiation signals, even in the presence of hematopoietic growth factors such as erythropoietin. Lineage commitment and differentiation may also be affected by altered expression of transcription factors involved in erythroid and megakaryocytic development, including GATA2 [133,134].

Although ineffective erythropoiesis in AA is primarily attributed to the immune-mediated depletion and dysfunction of HSPCs and erythroid progenitors, alterations in autophagic flux and mitochondrial quality control could potentially further compromise erythroid differentiation. In this context, the inflammatory and oxidative environment characteristic of AA may interfere with the autophagic mechanisms required for efficient mitochondrial remodeling during terminal erythropoiesis.

Taken together, current evidence supports a model in which autophagy and mitophagy may act as important modifiers of HSPC and erythroid cell fitness in AA, rather than being established primary drivers of disease pathogenesis. The interplay between chronic immune activation, cytokine signaling, oxidative stress, mitochondrial dysfunction, and autophagic quality control warrants further investigation. Defining whether autophagic defects are a cause or a consequence of HSPC injury—and determining whether they are reversible—will be essential for assessing the potential of autophagy-targeting strategies as therapeutic approaches in AA.

## 4. Therapeutic Targeting of Autophagy in Erythroid Disorders

Current treatments for congenital anemias and bone marrow failure syndromes primarily address disease manifestations rather than their underlying pathogenic mechanisms. These include splenectomy for splenomegaly, chronic blood transfusions to correct severe anemia, and iron chelation therapy to limit transfusion-associated iron overload [82]. Although curative approaches, such as hematopoietic stem cell transplantation and gene editing therapy, have emerged over the past decade [135,136]. Although substantial efforts have been made to improve the efficacy and reduce the cost of both HLA-haploidentical bone marrow transplantation [137,138] and non-myeloablative allogeneic HSCT [139], through optimized conditioning and reduced-intensity transplantation protocols, as well as through the implementation of educational and training programs [140], their high cost, technical complexity, and limited global accessibility continue to restrict their widespread implementation [141,142,143,144]. In this context, pharmacological modulation of autophagy and mitophagy has emerged as a promising and potentially more accessible strategy to restore erythroid homeostasis, improve erythroid precursor survival, and reduce oxidative stress across a broad spectrum of disorders, including myelodysplastic syndromes (MDS), Diamond–Blackfan anemia (DBA), Fanconi anemia (FA), sickle cell disease (SCD), and β-thalassemia. The clinical success of these approaches will depend on the precise identification of disease-specific autophagic defects and the selection of molecular targets capable of restoring cellular quality-control mechanisms (Table 1).

Among the most extensively investigated strategies is pharmacological inhibition of mTOR using rapamycin (sirolimus) and its derivatives (“rapalogs”), which constitute the first generation of mTOR-targeting compounds [149]. The translational relevance of this pathway is supported by the constitutive activation of PI3K/AKT/mTOR signaling observed in several hematological disorders, including MDS, β-thalassemia, and SCD [75,149]. In murine models of β-thalassemia and SCD, sirolimus improved anemia, reduced organ damage, and promoted ULK1-dependent autophagic clearance of excess α-globin [84]. In parallel, sirolimus also enhanced γ-globin expression and HbF production [40,91]. These preclinical findings have been corroborated by clinical studies demonstrating increased ULK1 expression, enhanced autophagy, and reduced α-globin accumulation in erythroid precursor cells from β-thalassemia patients receiving low-dose sirolimus [93]. Collectively, these results contributed to the Orphan Drug Designation granted by both the European Medicines Agency (EMA) and the U.S. Food and Drug Administration (FDA) for sirolimus in β-thalassemia and SCD [145]. Beyond classical rapalogs, second-generation dual mTORC1/mTORC2 inhibitors such as MLN0128 (INK128) have also shown therapeutic benefit in murine SCD models, suggesting that broader inhibition of mTOR signaling may provide additional advantages [40]. However, it is noteworthy that rapalogs remain at the preclinical stage of development for these erythroid disorders. In MDS, hypomethylating agents such as azacitidine may further complement these effects by restoring autophagy-mediated clearance of defective hematopoietic progenitors [150].

Autophagy also exerts a critical cytoprotective role in β-thalassemia through its coordinated interaction with other protein quality-control systems, including the ubiquitin–proteasome system (UPS) and heat shock proteins [86,94]. Whereas the UPS preferentially degrades soluble α-globin species, autophagy mediates the clearance of insoluble α-globin aggregates, thereby limiting intracellular toxicity [94]. Consistent with this complementary role, ULK1 deficiency exacerbates ineffective erythropoiesis and α-globin accumulation, whereas rapamycin treatment improves hemoglobin concentration, red blood cell count, and splenomegaly in β-thalassemic mice [84,151]. Similarly, in aplastic anemia (AA), where defective autophagy sensitizes CD34^+^ hematopoietic cells to apoptosis, pharmacological activation of autophagic flux may preserve the hematopoietic stem cell compartment and improve cell survival [152]. Notably, the clinical translation of mTOR-targeted regimens in severe AA remains under investigation, as illustrated by the clinical evaluation of sirolimus in refractory bone marrow failure settings (NCT02979873/17-H-0019).

Despite these encouraging results, chronic mTOR inhibition may cause systemic toxicity and immunosuppression, potentially increasing susceptibility to opportunistic infections by suppressing effector T-cell proliferation while promoting regulatory T-cell differentiation. These concerns have prompted the development of mTOR-independent therapeutic strategies [153,154]. Importantly, the consequences of sustained mTORC1 inhibition may extend beyond immune dysfunction and affect hematopoietic homeostasis. In adult HSCs, basal autophagy and endogenous suppression of mTORC1 activity are essential for maintaining low mitochondrial activity, limiting oxidative stress, and preserving stem cell quiescence [155,156]. However, although low mTORC1 activity is required to prevent premature HSC exhaustion, sustained mTORC1 inhibition may also impair the metabolic activation necessary for HSCs to exit quiescence and proliferate in response to acute stress, thereby reducing their repopulation capacity and multilineage reconstitution efficiency [157].

The effects of mTORC1 inhibition on erythropoiesis are similarly stage-dependent. Active mTORC1 signaling is required during the early stages of erythroid differentiation to support mitochondrial biogenesis, protein synthesis, and expansion of lineage-committed precursors [158]. Consequently, premature or systemic mTORC1 inhibition may impair mitochondrial biogenesis and early precursor expansion, resulting in microcytic anemia, erythroid hypoplasia, and cell-cycle arrest before cells reach the terminal stages at which organelle clearance becomes essential [158,159]. Mechanistically, systemic mTORC1 suppression promotes a shift from anabolic growth toward catabolic processes, affecting TOP mRNA translation, ribosome biogenesis, lipogenesis, and glycolysis, and potentially uncoupling heme biosynthesis from the protein synthesis required for erythroid maturation [158,160].

Thus, these observations highlight an important therapeutic limitation: although mTOR inhibition can stimulate autophagy and promote mitochondrial clearance, its effects are highly dependent on cell type and developmental stage. Broad or prolonged mTORC1 inhibition may therefore interfere with the anabolic and metabolic programs required for early hematopoietic and erythroid differentiation. This provides a strong rationale for developing more selective approaches that activate autophagy or mitophagy specifically at the appropriate stage of erythroid maturation, while minimizing disruption of mTOR-dependent physiological functions.

Small-molecule enhancers of rapamycin (SMERs), such as SMER28, stimulate autophagy through ATG5-dependent mechanisms independently of mTOR signaling [147]. In DBA, SMER28 significantly enhanced erythroid differentiation and globin gene expression, suggesting a promising therapeutic alternative with potentially fewer adverse effects than corticosteroids or rapamycin [112]. Natural compounds have also attracted attention. For example, resveratrol activates autophagy through SIRT1 signaling and partially corrects hematopoietic defects in FA by reducing oxidative stress and improving erythroid cell survival [146].

Targeting mitophagy has emerged as another particularly attractive strategy for disorders characterized by mitochondrial dysfunction and oxidative stress. In SCD, mitochondrial retention contributes to excessive ROS production, chronic inflammation, and cellular injury [147]. Both sirolimus and RN-1, a pharmacological LSD1 inhibitor, reduce mitochondrial retention and oxidative stress, likely through epigenetic activation of pro-mitophagy genes such as ATG7 [75]. The importance of mitochondrial quality control is further illustrated in FA, where FANCC functions as a selective autophagy receptor involved in mitophagy regulation [161]. Likewise, in X-linked sideroblastic anemia, therapeutic modulation of the AMPK/mTOR pathway or direct targeting of mitophagy receptors such as BNIP3L/NIX and PINK1/Parkin may facilitate the elimination of dysfunctional or iron-overloaded mitochondria while preserving cellular metabolism [162,163].

The therapeutic potential of modulating mitochondrial quality control and protein synthesis in sideroblastic anemias is further underscored by recent findings demonstrating that mitochondrial tRNA pseudo uridylation and mTOR signaling directly regulate erythropoiesis [148]. In Pearson syndrome, another disorder characterized by mitochondrial dysfunction and sideroblastic anemia, restoration of mitochondrial quality-control pathways may similarly represent a promising therapeutic strategy [120]. Importantly, translational efforts aimed at enhancing mitochondrial quality control and metabolic homeostasis in pediatric primary mitochondrial diseases, including Pearson syndrome, are currently under clinical investigation (NCT03384420).

At the molecular level, ULK1 has emerged as a master regulator of erythroid autophagy and an attractive therapeutic target [84]. In parallel, microRNAs (miRNAs) have gained increasing translational interest because they regulate the expression of key autophagy-related genes, including ULK1, ATG5, ATG7, and BECN1 [151]. Notably, disruption of the miR-144/451 cluster in murine β-thalassemia models reduced mTORC1 activity, enhanced ULK1-dependent autophagy, improved erythroid survival, and decreased α-globin accumulation [89]. These findings open new perspectives for epigenetic and RNA-based therapies aimed at selectively modulating erythroid autophagy.

Future therapeutic strategies will likely combine autophagy or mitophagy activators with antioxidant therapies, iron chelators, and metabolic modulators. Such combinatorial approaches may be particularly beneficial in transfusion-dependent β-thalassemia, in which iron overload exacerbates oxidative stress and further disrupts erythroid homeostasis [151]. Because autophagy also contributes to intracellular iron homeostasis through ferritinophagy, understanding the interplay between iron metabolism and autophagic pathways will be essential for optimizing these combination therapies.

The development of such interventions must, however, take into account the stage-dependent role of mTOR signaling. Whereas attenuated mTORC1 activity is required to preserve long-term HSC quiescence and self-renewal, increased mTORC1 activity promotes the metabolic expansion of lineage-committed erythroid progenitors [158,164,165]. Thus, excessive activation of autophagy or indiscriminate inhibition of mTORC1 could disrupt the balance between stem cell preservation and progenitor expansion [164]. Moreover, excessive autophagy activation may itself promote apoptosis, cytotoxicity, or stem cell exhaustion. A deeper understanding of the interplay between autophagy, apoptosis, and other protein quality-control pathways will therefore be essential to define safe therapeutic windows.

Overall, transient, stage-specific, or cell-targeted modulation of autophagy and mitophagy, rather than sustained systemic activation or mTORC1 inhibition, may represent a more appropriate therapeutic strategy. Such approaches could promote mitochondrial and protein quality control during the stages in which these processes are required, while preserving the mTOR-dependent anabolic programs necessary for HSC maintenance and erythroid progenitor expansion.

## 5. Conclusions and Perspectives

In erythroid disorders, dysregulated autophagy and mitochondrial retention are recurrent findings, but they do not necessarily reflect the same underlying pathological mechanism. Residual mitochondria may be damaged, remain metabolically active, or represent a functionally heterogeneous population. Their persistence may directly exacerbate oxidative stress and membrane damage, arise secondarily from stress erythropoiesis or premature release of erythroid cells into the circulation, or reflect an adaptive response that has become insufficient. Similarly, an increased abundance of autophagosomes may indicate enhanced autophagic activity or, conversely, impaired autophagic flux. Mitochondrial retention should therefore be viewed as a convergent phenotype whose underlying mechanisms must be established in each disease, rather than as evidence of a uniform primary defect in mitophagy.

Future studies will need to determine precisely when, where, and why mitochondrial clearance fails. Analyses performed at clearly defined stages of erythroid differentiation, using primary human cells and patient-derived models, should combine dynamic measurements of autophagic and mitophagic flux with assessments of mitochondrial abundance and function, enucleation, membrane remodeling, cellular deformability, and red blood cell survival. It will also be important to determine how mitochondrial clearance is coordinated with heme and globin synthesis, iron homeostasis, and other cellular remodeling pathways, as well as how this intrinsic program is influenced by the bone marrow niche, stress erythropoiesis, entry into the circulation, and splenic maturation. Such approaches should help distinguish disease-specific mechanisms from the shared consequences of ineffective erythropoiesis and ultimately identify biologically meaningful patient subgroups.

Pharmacological studies have already demonstrated that autophagy-related pathways can be modulated therapeutically. However, because currently available compounds often affect multiple cellular processes, these studies do not establish that mitophagy is their sole or primary therapeutically relevant target. The timing of intervention will also be critical: mitochondria are indispensable during the early stages of erythropoiesis, whereas their removal becomes essential during terminal maturation. Effective therapeutic strategies will therefore need to restore the appropriate process in the relevant cells and at the correct stage of differentiation, rather than indiscriminately stimulating autophagy. The central challenge is now to understand the regulatory switch that transforms mitochondria from organelles essential for the biosynthetic and metabolic functions of erythroblasts into organelles whose persistence compromises the functional properties of mature red blood cells—and to determine how this transition can be safely restored when it is disrupted by disease.

## Figures and Tables

**Figure 1:**
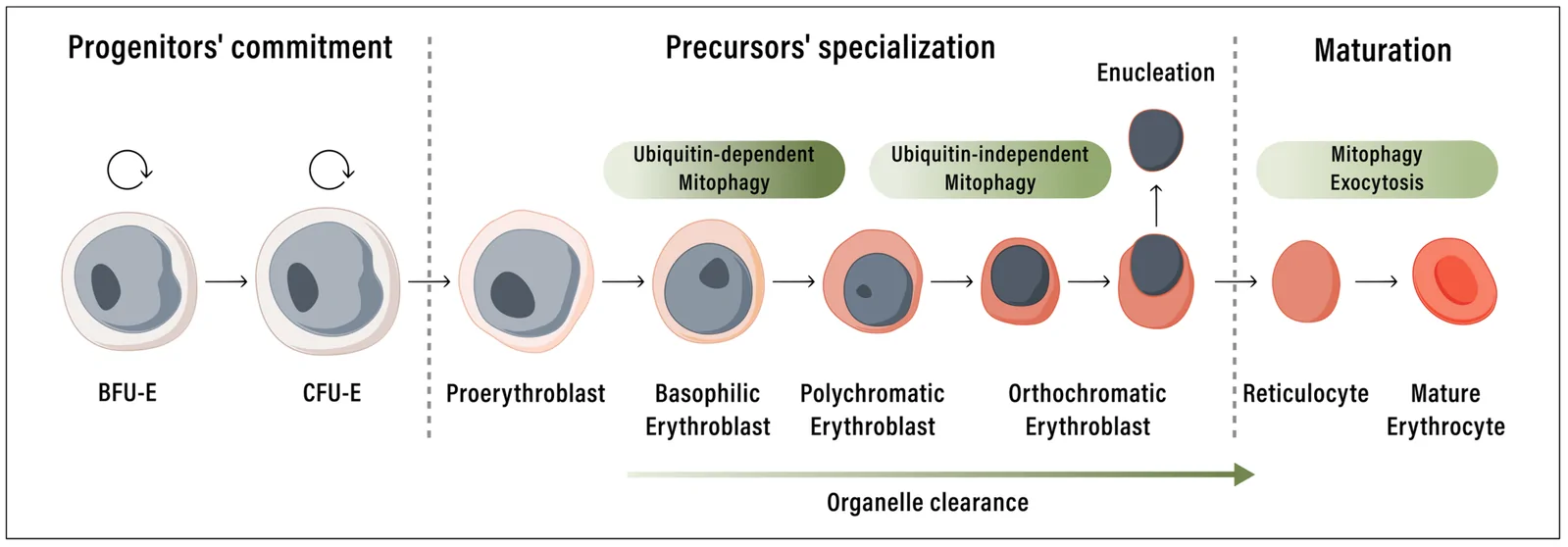
Erythropoiesis, from progenitor cell commitment to erythrocyte maturation. Erythroid progenitors (BFU-E, Burst-Forming Unit–Erythroid, and CFU-E, Colony-Forming Unit–Erythroid) undergo several rounds of proliferation before committing to differentiation into proerythroblasts. Erythroid precursors then gradually differentiate into basophilic, polychromatophilic, and finally orthochromatic erythroblasts. Throughout this process, cell size progressively decreases, chromatin condenses, hemoglobin synthesis increases, and intracellular organelles are progressively cleared. Terminal differentiation culminates in enucleation, generating a reticulocyte. Following the clearance of residual organelles, reticulocytes are released into the bloodstream, where they complete their maturation into erythrocytes. Green boxes indicate main mitophagy pathway at different differentiation steps. Created with BioRender.com.

**Figure 2:**
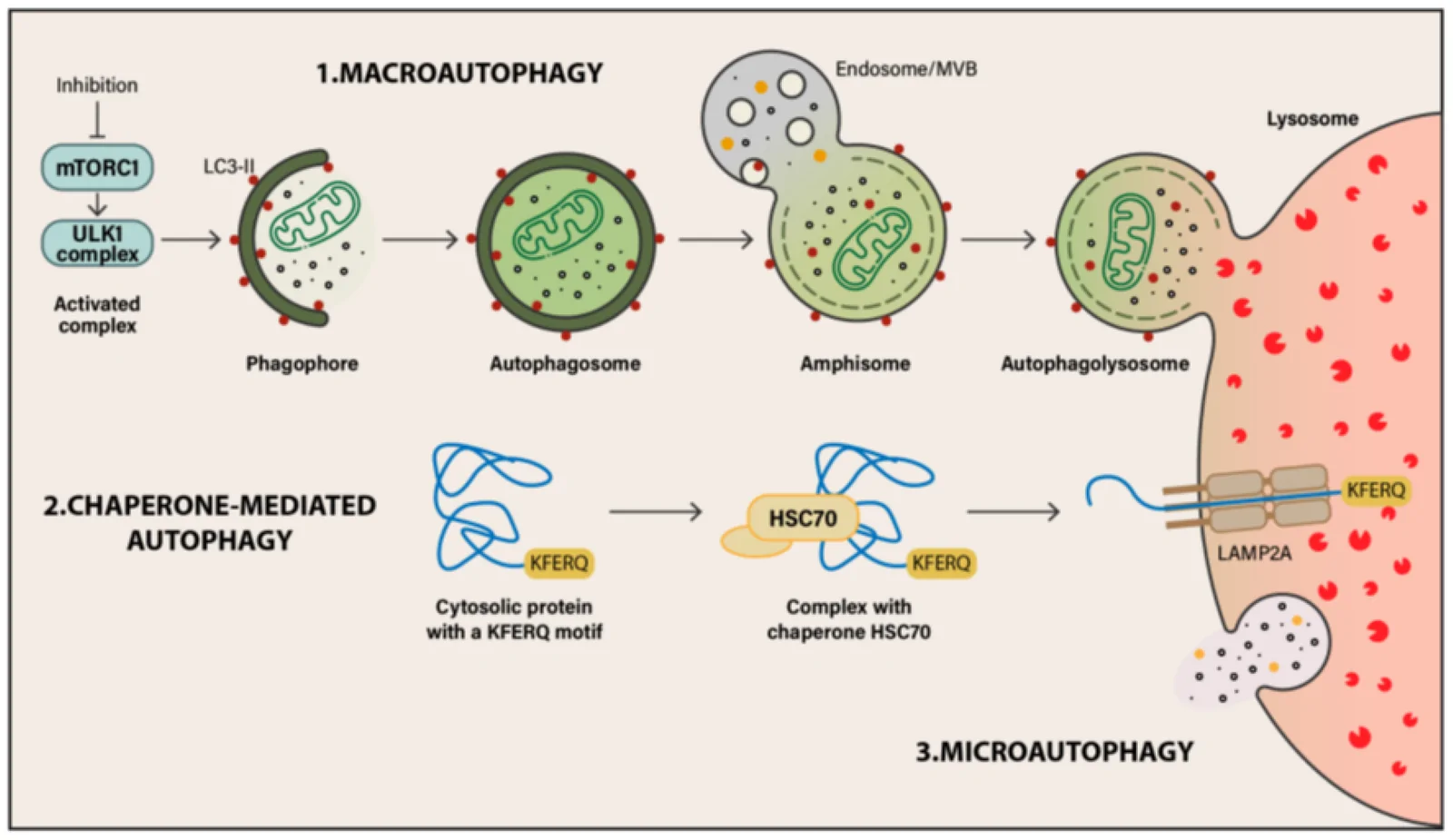
Schematic illustration of the three major autophagic pathways. Top: (1) Macroautophagy initiation begins with mTORC1 inhibition, leading to the activation of the ULK1 complex. Phagophore nucleation and elongation mediated by LC3-II lipidation engulf intracellular cargo (such as mitochondria) to form an autophagosome. The autophagosome can fuse with late endosomes or multivesicular bodies (MVBs) to form an amphisome, which subsequently fuses with a lysosome to form an autophagolysosome for enzymatic degradation. Bottom Left: (2) Chaperone-Mediated Autophagy (CMA) involves the selective recognition of cytosolic proteins containing a KFERQ-like motif by the HSC70 chaperone complex, followed by direct translocation into the lysosomal lumen through the LAMP2A receptor complex. Bottom Right: (3) Microautophagy occurs via direct invagination of the lysosomal membrane to sequester cytosolic components. Created with BioRender.com.

**Figure 3:**
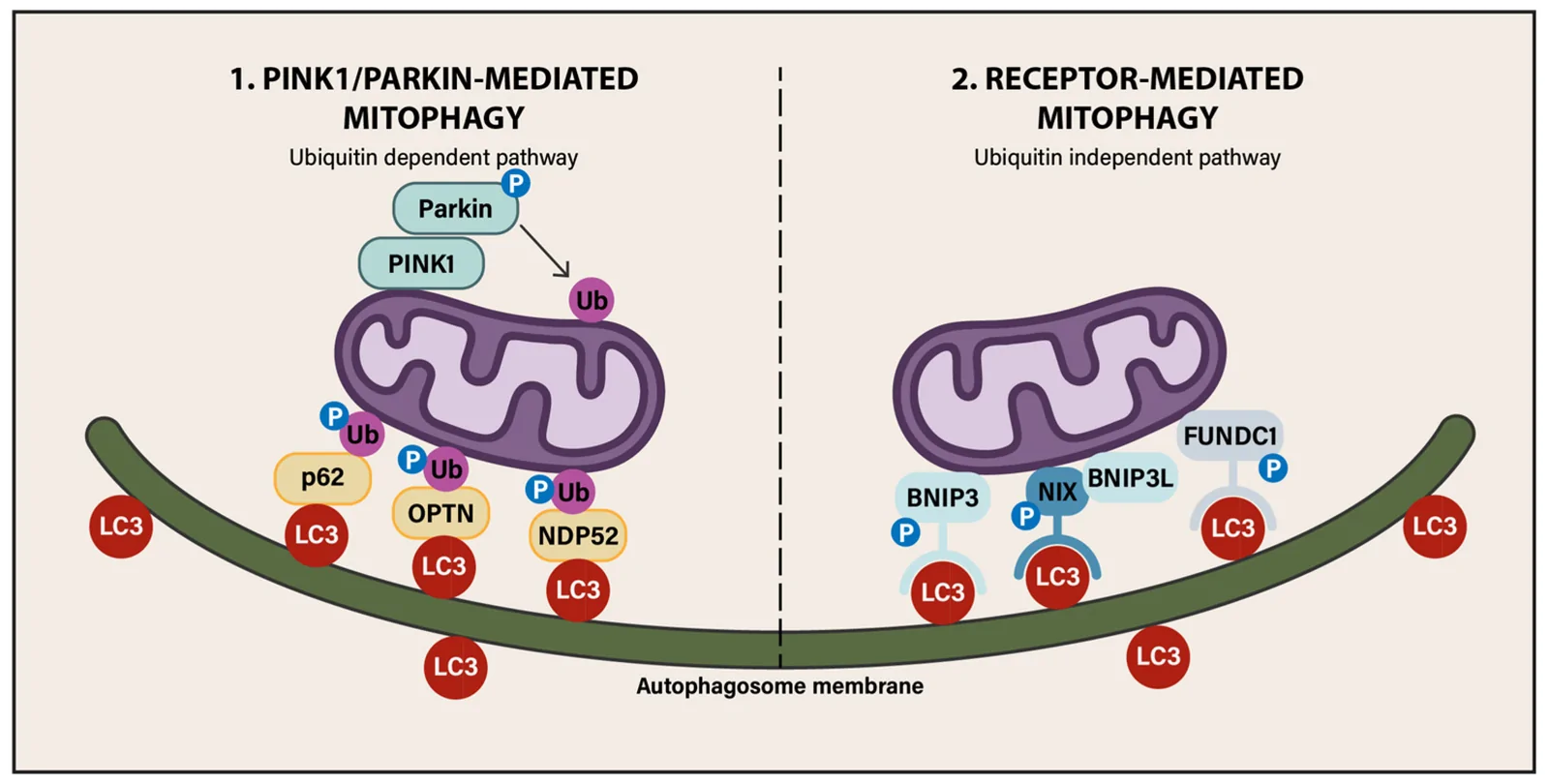
Schematic illustration of the two major mitophagic pathways. Left: (1) PINK1/Parkin-mediated mitophagy: Following mitochondrial depolarization, stabilized PINK1 phosphorylates both OMM-bound ubiquitin (Ser65) and Parkin, establishing a feed-forward ubiquitination loop. Phosphorylated ubiquitin chains preferentially recruit the primary autophagic adaptors optineurin (OPTN) and NDP52, which interact with LC3 to initiate phagophore assembly, while p62/SQSTM1 predominantly promotes mitochondrial clustering. Right: (2) Receptor-mediated mitophagy: Damaged or stressed mitochondria express outer membrane mitophagy receptors (such as BNIP3, NIX, or FUNDC1) that directly bind LC3 via their LIR motifs to recruit autophagosomal membranes independently of ubiquitin. Created with BioRender.com.

**Table 1:** Autophagy and mitophagy alterations in erythroid diseases and their potential therapeutic implications. This table summarizes the main autophagy- and mitophagy-related defects associated with mitochondrial retention, oxidative stress, and ineffective erythropoiesis across different erythroid disorders discussed in this review, as well as the principal therapeutic strategies and molecular targets currently under investigation. It also highlights potential drugs in development and pharmacological compounds currently under preclinical or clinical investigation.

Disease	Autophagy/Mitophagy Defect	Therapeutic Strategy	Key Molecular Targets	Potential Drugs/Developing Agents (Status/Stage)	References
**Sickle cell** **disease (SCD)**	Mitochondrial retention, increased mtDNA, persistent oxidative metabolism, ROS accumulation, reduced NIX/PINK1 expression	Enhance autophagy and mitophagy to eliminate retained mitochondria and reduce oxidative stress	NIX, PINK1, ATG7, mTOR, LSD1	Sirolimus/Rapamycin (FDA/EMA Orphan Drug, Phase II), RN-1 (LSD1 inhibitor, Preclinical), MLN0128/INK128 (mTORC1/2 inhibitor, Preclinical)	[75,76,77,78,79,80,145]
**β-Thalassemia**	Excess α-globin aggregates, oxidative stress, ineffective erythropoiesis, impaired mitochondrial clearance despite increased autophagy signaling	Stimulate ULK1-dependent autophagy and mitophagy to promote α-globin degradation and erythroid maturation	mTOR, ULK1, LC3, BNIP3L/NIX, PINK1	Sirolimus/Rapamycin (FDA/EMA Orphan Drug, Clinical trials), AZD8055 (mTORC1/2 inhibitor, Preclinical), Chloroquine (Autophagy flux modulator, Preclinical/Clinical)	[81,82,83,84,85,86,89,90,91,92,93,94,95,96,145]
**Myelodysplastic syndromes (MDS)**	Defective NIX-mediated mitophagy, reduced LC3 activation, impaired autophagosome formation, mitochondrial accumulation	Restore autophagic and mitophagic flux to improve mitochondrial clearance	mTOR, NIX (BNIP3L), LC3	Azacitidine (Hypomethylating agent, Approved/Clinical), mTOR inhibitors/Rapalogs (Preclinical/Phase I-II)	[97,98,99,100,101]
**Fanconi** **anemia (FA)**	Defective DNA repair associated with impaired selective mitophagy and mitochondrial quality control	Activate autophagy and mitophagy to restore mitochondrial homeostasis	FANCC, FANCL, SIRT1	Resveratrol (SIRT1 activator, Preclinical), Rapamycin (Preclinical)	[102,103,104,105,106,107,146]
**Diamond Blackfan** **anemia (DBA)**	Ribosomal dysfunction with impaired erythroid differentiation and altered autophagy	mTOR-independent activation of autophagy to rescue erythropoiesis	ATG5	SMER28 (Preclinical)	[112,147]
**X-linked sideroblastic anemia**	ALAS2 mutations causing defective heme synthesis, iron-loaded mitochondria, impaired mitophagy	Restore mitochondrial quality control and promote clearance of iron-overloaded mitochondria	ALAS2, GATA1, BNIP3L/NIX, PINK1/Parkin, AMPK/mTOR	Sirolimus/Rapamycin (Preclinical)	[113,114,115,116,117,148]
**Pearson** **syndrome**	Mitochondrial dysfunction, impaired mitophagy, mitochondrial persistence, oxidative stress	Restore mitophagy and mitochondrial quality control	Mitophagy regulators, mitochondrial quality-control pathways	Autologous CD34+ Cells enriched with blood derived mitochondria (Clinical trial NCT03384420)	[115,116,117]
**Aplastic** **anemia (AA)**	Oxidative stress, mitochondrial dysfunction, impaired autophagy, defective erythropoiesis	Pharmacological induction of autophagy to preserve hematopoietic stem cell survival	CD34^+^ cells, autophagy activators, mTOR modulation	Sirolimus/Rapamycin (Clinical trial, Phase II NCT02979873)	[118,119,120,121,122,123,124,125]

## Data Availability

No new data were created or analyzed in this study. Data sharing is not applicable to this article.

## Abbreviations

The following abbreviations are used in this manuscript:

15-LOX	15-Lipoxygenase
AA	Aplastic Anemia
ATG	Autophagy Related Genes
BasoE	Basophilic Erythroblast
BM-EPCs	Bone marrow endothelial progenitor cells
CCCP	Carbonyl cyanide m-chlorophenylhydrazone
CMPs	Common myeloid progenitor cells
CTLs	Cytotoxic T lymphocytes
DBA	Diamond–Blackfan Anemia
ER	Endoplasmic reticulum
FA	Fanconi Anemia
GPA	Glycophorin A
HbF	Fetal Hemoglobin
HPSC	Hematopoietic stem and progenitor cell
IFN-γ	Interferon γ
iNOS	Inducible Nitic Oxide Synthase
LIR	LC3-interacting region
MDS	Myelodysplastic Syndromes
MDS-RS	Myelodysplastic Syndromes with ring sideroblasts
MPPs	Multipotent progenitor cells
mTORC1	Mammalian target of rapamycin complex 1
MVBs	Multivesicular Bodies
OPTN	Optineurin
OrthoE	Orthochromatic Erythroblast
PHB2	Prohibitin-2
PI3P	Phosphatidylinositol-3-phosphate
ProE	Pro Erythroblast
RBC	Red Blood Cell
ROS	Reactive Oxygen Species
SCD	Sickle Cell Disease
SMER	Small-molecule enhancers of rapamycin
TCA	Tricarboxylic Acid
TfR/CD71	Transferrin Receptor
TH1	T helper 1 cells
TNFα	Tumor necrosis factor α
UPR	Unfolding Protein Response
UPS	Ubiquitin Proteasome System
XLSA	X-linked sideroblastic anemia
